# Sustainable Cauliflower-Patterned CuFe_2_O_4_ Electrode Production from Chalcopyrite for Supercapacitor Applications

**DOI:** 10.3390/nano13061105

**Published:** 2023-03-20

**Authors:** Moctar Mbebou, Safa Polat, Huseyin Zengin

**Affiliations:** 1 Material Research and Development Centre, Karabuk University, Karabuk 78050, Turkey; 2 Nano Energy Laboratory, Karabuk University, Karabuk 78050, Turkey; 3 Metallurgy and Materials Engineering, Karabuk University, Karabuk 78050, Turkey; 4 Institute of Chemical Technology of Inorganic Materials (TIM), Johannes Kepler University Linz, 4040 Linz, Austria

**Keywords:** chalcopyrite, leaching, CuFe_2_O_4_, electrode, supercapacitor

## Abstract

The primary purpose of this study was to produce an ore-based high-capacity supercapacitor electrode. For this, chalcopyrite ore was first leached with nitric acid, and then metal oxide synthesis was carried out immediately on nickel foam using a hydrothermal technique from the solution. Cauliflower-patterned CuFe_2_O_4_ with a wall thickness of about 23 nm was synthesized on the Ni foam surface, characterized by XRD, FTIR, XPS, SEM, and TEM investigations. The produced electrode also displayed a feature of a battery-like charge storage mechanism with a specific capacity of 525 mF cm^−2^ at 2 mA cm^−2^ current density, energy of 8.9 mWh cm^−2^, and a power density of 233 mW cm^−2^. Additionally, even after 1350 cycles, this electrode still performed at 109% of its original capacity. The performance of this finding is 255% higher than that of the CuFe_2_O_4_ in our earlier investigation; despite being pure, it performs far better than some of its equivalents in the literature. Obtaining such performance from an electrode made from ore indicates that the use of ore has a lot of potential for supercapacitor production and property improvement.

## 1. Introduction

Recent years have seen a growth in energy consumption, mostly as a result of technological developments connected with society’s quest for comfort [1]. This situation is leading to a continued reduction in non-renewable coal and oil resources [2,3,4]. Consequently, there is a great demand for energy storage systems that are environmentally friendly, practical, accessible, and capable of supplying a consistent supply of energy in a short period of time [5]. In this regard, a huge amount of scientific research is now being carried out to investigate various possibilities that might store energy in a more straightforward and expedient manner, in greater amounts, and for longer durations [6]. Because of their excellent power efficiency, quick charge/discharge ability, and extended rate capability, supercapacitors (SCs) have become one of the most prevalent types of energy storage devices (ESD) and are currently in the process of being developed [7]. SCs are divided into two categories, electrochemical double-layer capacitors (EDLCs) and pseudocapacitors (PCs), based on how they store their energy [8]. EDLCs store energy electrostatically by the neutron absorption of surface particles at the conductive interfaces, while PCs store energy through rapid and reversible Faradic surface interactions between electrolyte ions and electroactive materials [9]. The electrochemical characteristics of standard EDLCs are poorer, but their cycle life is much longer than those of pseudocapacitors. It was reported that hybrid materials that comprise both PCs and EDLCs are superior in terms of both cycle life and high conductivity [10].

Spinel ferrites (SF) MFe_2_O_4_ (M = Cu, Mn, Ni, Zn, etc.) are frequently utilized as electroactive anode materials for PCs in this context due to their capacity to display many redox states as well as their electrochemical stability [11,12,13,14,15]. CuFe_2_O_4_ stands out among them as a highly qualified candidate for an anode material due to its high theoretical capacity (895 mAh g^−1^) [16], low band gap (1.7–1.9 eV) [17], nontoxicity, room-temperature stability [16,18], environmental friendliness, and advantages of natural occurrence [11,13]. CuFe_2_O_4_ is so environmentally friendly that it can be directly synthesized synthetically from chalcopyrite ore, a significant natural supply of copper (Cu) and iron (Fe). It is desirable for such structures to have a particularly large surface area and sometimes a special geometry for high-capacity energy storage in devices such as supercapacitors [19,20]. In this regard, the hydrothermal approach can be selected since it has shown itself in several experiments to directly synthesize such nano-sized structures with a large surface area and unique geometry on a current collector surface at low temperatures [21,22,23]. It is possible to extract copper and iron from chalcopyrite by the hydrometallurgical method with different acids [24].

In this work, we intended to ascertain the greatest copper and iron content from chalcopyrite using varied concentrations of HCl, HNO_3_, and H_2_SO_4_ while considering each of the hypotheses given in this study. Then, a cauliflower-patterned CuFe_2_O_4_ was produced using this solution with the highest concentration on the current collector nickel foam surface using the hydrothermal process. The characterization of the products was examined in detail with XRD, FTIR, XPS, SEM, TEM and BET analyses. Their electrochemical performances were investigated via cyclic voltammetry (CV), galvanostatic charge–discharge (GCD) and impedance (EIS) measurements. Finally, the originality of this study and its place in the literature was determined by comparing the results with the literature.

## 2. Experimental

### 2.1. Materials and Methods

Chalcopyrite ore enriched by flotation in a pilot-sized ore preparation plant in the Hanönü district of the Kastamonu province of Turkey was used in this investigation as a metal source. The chemical compositions of the concentrated chalcopyrite were measured using wave-length dispersive X-ray fluorescence (XRF), and the results are given in Table 1 [25]. The extraction process involved the use of hydrochloric acid (HCl, 37% *w*/*w*), nitric acid (HNO_3_, 68% *w*/*w*) and sulfuric (H_2_SO_4_, 95–98% *w*/*w*). The metal oxide synthesis was carried out on nickel foam substrate in an autoclave with a Teflon-lined stainless steel body.

### 2.2. Leaching of Chalcopyrite

A 250 mL beaker containing 3 g of chalcopyrite and 50 mL of deionized water was agitated at 300 rpm for 30 min at room temperature as part of the leaching process. Then, this leaching solution was first filtered with Watman filter paper and then obtained by centrifugation at 3000 rpm. HCl, HNO_3_ and H_2_SO_4_ acids were also added to the solution at various rates in order to test the impact on copper and iron extraction in these methods. The concentrations of these solutions were adjusted to 10, 20, 30, and 40 ppm by varying the amount of acid. To measure the concentration, each solution was used separately in the leaching process.

### 2.3. Electrode Preparation by Hydrothermal Process

**Figure 1:**
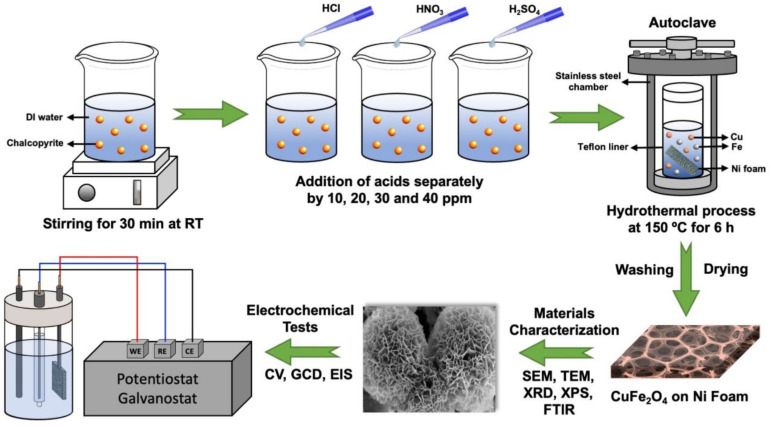
Schematic illustration of the production and characterizations.

For the preparation of the electrodes, the leaching solution with the maximum concentration of copper and iron was employed. The Teflon lining was first filled with 3 mL of the solution. About 10 mg of urea was then added to this solution. Deionized water was then added until the total volume of the solution reached 20 mL. Then, a piece of nickel foam cut in 2 × 3 cm^2^ dimensions was added to the solution and the lid was tightly closed. The Teflon was placed in a stainless steel autoclave and then transferred to the furnace. It was kept in the furnace at 150 °C for 6 h and then allowed to cool to room temperature. After cooling, the autoclave cover was opened, the nickel foam inside was taken, and the surface was washed with distilled water and dried in an oven at 60 °C for a day on a watch glass. The electrode production steps are schematically demonstrated in Figure 1.

### 2.4. Materials Characterizations

The concentrations of copper and iron in the solutions were measured using atomic absorption spectrometers (AAS, Thermo Scientific ICE 3400, USA). The crystallographic analyses of the products were carried out via X-ray diffraction (XRD, Rigaku Ultima IV X-ray diffraction, Japan) with Cu-based Ka radiation at a wavelength of 0.1546 nm. Fourier transform infrared spectroscopy (Bruker Alpha, Germany) was used to determine the chemical bond structure, with a resolution of 2 cm^−1^ at wave numbers between 400 and 4000 cm^−1^. X-ray photoelectron spectroscopy (XPS, Flex Mod System, Germany) was used to examine the electronic structure and valence states of each component using a monochromatic Al X-ray source (Al-K line: 1486.6 eV). A high-resolution transmission electron microscope (HR-TEM, Thermo Scientific Talos F200S, USA) and a scanning electron microscope (SEM, Carl Zeiss Ultra Plus, Germany) were used to analyze the microstructure and morphology of the structures.

### 2.5. Electrochemical Measurement

The electrochemical performances of hydrothermally manufactured electrodes were investigated with a potentiostat (Ametek Parstat 4000, USA) via cyclic voltammetry (CV), galvanostatic charge–discharge (GCD) and electrochemical impedance spectroscopy (EIS) measurements. All electrochemical experiments were performed using a three-electrode setup. In this system, a sample created via the hydrothermal process served as the working electrode, while a graphite rod and Ag/AgCl (3 M KCl) served as the counter and reference electrodes, respectively. The electrolyte liquid was a 6 M KOH solution made with deionized water (18.25 Mohm). A portion of these nickel foams, measuring 0.5 × 1.5 cm^2^, was submerged in the electrolyte after being compressed with a pressure of around 10 MPa. While GCD measurements were carried out at a current density of 1–32 mA cm^−2^, CV measurements were conducted in the potential range of 0 to 0.45 V. Additionally, EIS measurements were made in the frequency range of 100 kHz to 0.1 Hz with a bias potential of 0 V and a signal amplitude of 10 mV AC. The specific capacitance (Cs) of the electrodes was computed by following the nonlinear discharge curves [26].

(1) $$Cs=\frac{I\times ∆t}{∆V\times S}$$

In this equation, Cs is the area capacitance in millifarads (mF), I is the discharge current constant in milliamperes (mA), t is the discharge duration in seconds (s), V is the potential window in volts (V), and S is the surface area of the working electrode in square centimeters (cm^2^). In addition, based on the information gathered from the Equation (1), the energy (E) and power (P) densities of the electrode were calculated using Equations (2) and (3) [26].

(2) $$E=\frac{Cs\times V^{2}}{7.2}$$

(3) $$P=\frac{3600\times E}{∆t}$$

## 3. Results and Discussion

### 3.1. Material Characterization

**Figure 2:**
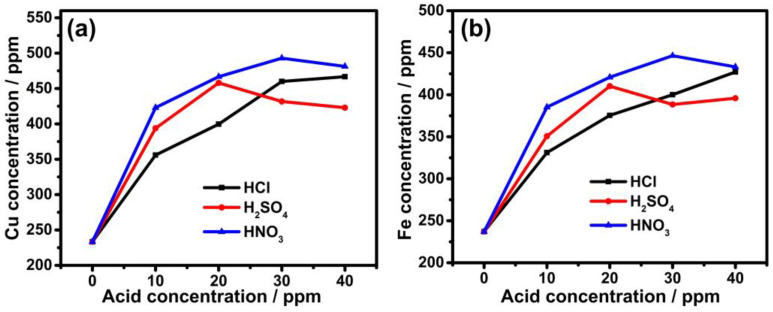
Concentrations of (**a**) copper and (**b**) iron in different acid concentrations.

Figure 2 displays the copper and iron concentrations in acid-prepared leaching solutions. These findings show that the copper and iron concentrations in pure water solutions were detected as 230 ppm. Iron concentrations were 350, 375, 400, and 427 ppm, respectively, whereas the concentrations of copper were 330, 400, 460, and 466 ppm in 10, 20, 30, and 40 ppm HCl solutions, respectively. Moreover, when H_2_SO_4_ was added in the same order, the concentrations of copper were around 393, 457, 431, and 422 ppm, whereas iron concentrations were measured as 350, 410, 388, and 395 ppm, respectively. Adding HNO_3_ in the same order slightly increased the concentrations to 385, 420, 446, and 433 ppm for iron and 422, 466, 492, and 481 ppm for copper, respectively. The better leaching performance of nitric acid solution can be attributed to the improved oxidation capacity of nitrate ions, which increases the dissolution of ores by acting as a strong oxidant due to the formation of O, NO and NO_2_ gases [27]. These findings show that a 40 ppm HNO_3_ solution produced the highest concentrations of both copper and iron.

**Figure 3:**
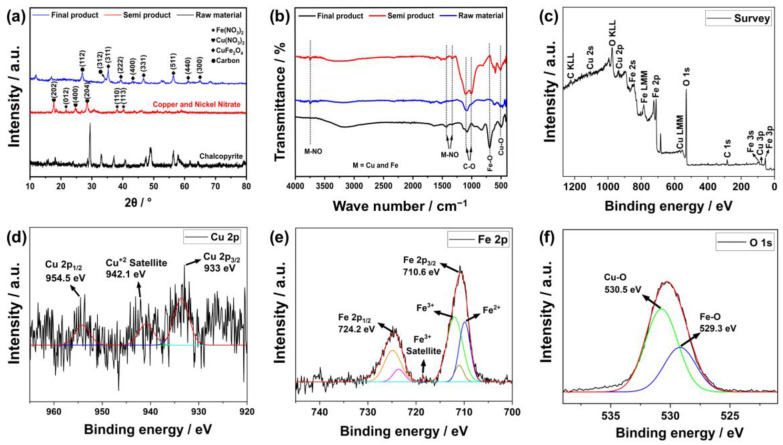
Characterization results of the products via (**a**) XRD, (**b**) FTIR, (**c**) XPS survey scanning and (**d**–**f**) XPS special scanning.

The characterizations of the products in this study were carried out via XRD and FTIR analyses as raw materials, semi-products and final products. The XRD results are given in Figure 3a. These findings show that the peaks at roughly 30°, 49° and 57° 2θ in the XRD graph of the raw material originate from the (204), (331) and (511) planes of chalcopyrite [28]. Additionally, the semi-product contained the planes of copper nitrate (202), (400), and (204) at 18°, 24°, and 29° [29] as well as the planes of iron nitrate (012), (110) at 22°, 36°, and 39° [30]. The planes of copper ferrite (311), (222), (400), (331), (511), (440) and (300) at about 35°, 39°, 42°, 45°, 56°, 61° and 64° were detected in the hydrothermally created final electrode [31]. Similarly, the FTIR results are given in Figure 3b. According to these results, the low-intensity peaks observed at approximately 410–470 cm^−1^ were probably due to S-S bonds in the Cu-Fe-S system [32]. These were probably caused by the sulfur bonds in the CuS and FeS_2_ composition of chalcopyrite. On the other hand, vibration peaks for Cu-O and Fe-O bonds were observed at wave numbers of about 456 and 598 cm^−1^, respectively, which confirms the presence of the copper ferrite compound [33]. Other carbon bonds were probably due to urea residue and other impurities involved during production.

The final product was also subjected to XPS analysis in order to further verify the findings. Figure 3c shows the overview of this analysis. The sample content mostly consists of Cu, Fe, O and C components based on this result. A low amount of carbon was observed in this content, as evidenced by its severity, which is probably due to impurities and residual urea used in production. On the other hand, salient peaks for the 2p and 1 s orbitals of copper, iron and oxygen were observed, which are signs of chemical bonding. For further interpretation, specific scans of these elements are given in Figure 3d–f. In Figure 3d, some peaks were found at about 933, 942.1, and 954.5 eV corresponding to 2p_2/3_, +2 satellite and 2p_1/2_ orbitals of copper, respectively [34,35]. This indicates that copper is located in the octahedral and tetrahedral regions. Similarly, iron + 3 ion also has peaks corresponding to octahedral regions at 710.6 eV and tetrahedral regions at 724.2 eV [35]. On the other hand, the peaks at 529.3 and 530.5 eV observed at 1 s of oxygen probably indicate Cu–O and Fe–O bindings [36]. According to these findings, the iron and copper in the content create a metal oxide with a spinel structure.

**Figure 4:**
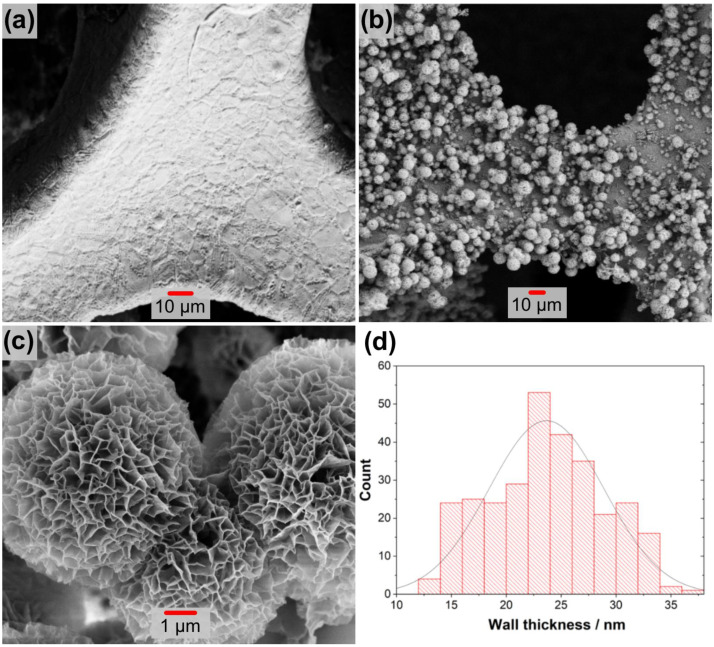
SEM images of the electrode: (**a**) pure Ni foam and (**b**,**c**) Ni foam image with different magnification after synthesis. (**d**) Wall size histogram graph.

**Figure 5:**
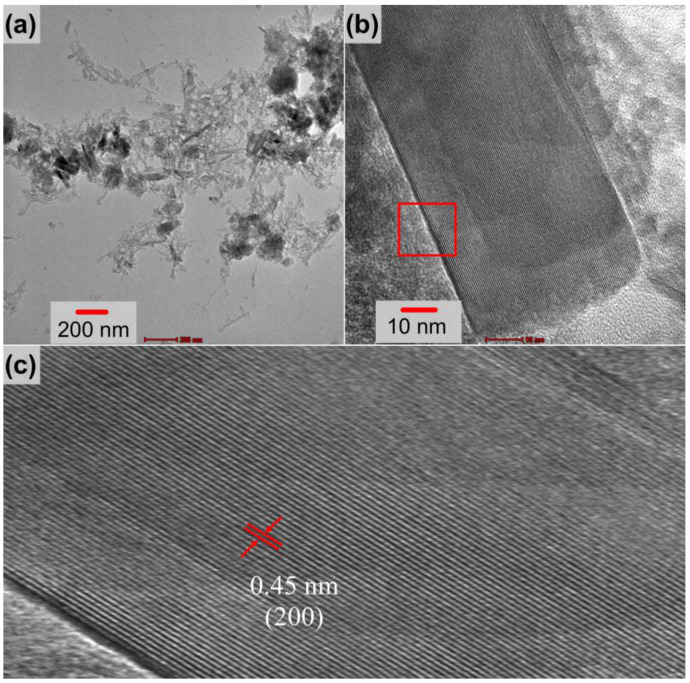
TEM images of the electrode (**a**) at low magnification and (**b**,**c**) as high-resolution images.

SEM and TEM analyses were performed to examine the microstructure and morphology of the metal oxide formations. The results are given in Figure 4. As shown in Figure 4a, the surface of untreated nickel foam used as a substrate was quite clear before hydrothermal synthesis. After the hydrothermal process, structures with various morphologies were formed as illustrated in Figure 4b,c. The foam surface seems to be completely covered with lumpy nodules. These nodules exhibit a rose or cauliflower appearance, as shown in Figure 4c. The average wall thickness distribution of this design was estimated using data from about 300 measurements and is shown in the histogram graph in Figure 4d. These data indicate that the thickness distribution ranges between 5 and 55 nm, with an average of about 23 nm. The morphology of these structures was examined with the TEM analysis given in Figure 5a–c. These formations are observed as partially stripy and partially platy, as shown in Figure 5a,b. This structure has an interplanetary distance of around 0.45 nm, which is based on the CuFe_2_O_4_ (200) plane, according to high-resolution images in Figure 5c.

### 3.2. Electrochemical Performance

**Figure 6:**
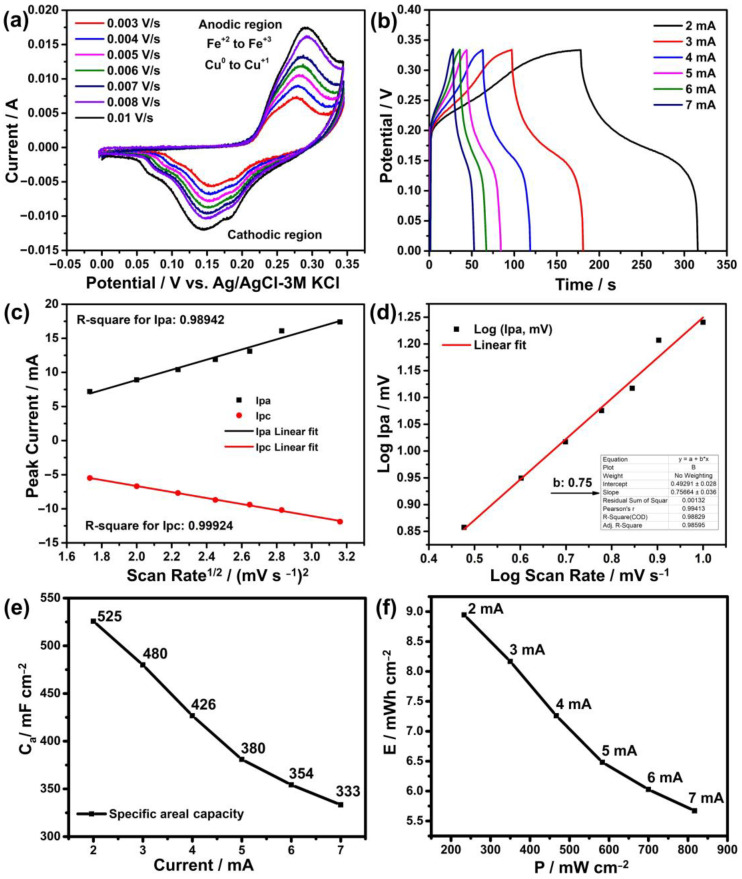
Electrochemical measurement results: (**a**) cyclic voltammetry, (**b**) galvanostatic charge–discharge, (**c**) Randel–Sevic, (**d**) electrochemical kinetics, (**e**) specific capacity and (**f**) energy vs. power density.

Cyclic voltammetry (CV) measurements were first carried out at various scanning speeds to examine the electrochemical performance of the electrodes produced using the hydrothermal technique. Graphic representations of the results are shown in Figure 6a. According to these findings, cathodic positive peaks can be seen in the range of 0.25 to 0.30 V, whereas anodic negative peaks are in the range of 0.15 to 0.065 V. These peaks are probably caused by the oxidation and reduction reactions of copper and iron ions with OH^−^ ions in the solution as in the following reaction [37].

CuFe_2_O_4_ + H_2_O + 2e^−^ ↔ CuO + 2FeO + 2OH^−^ (4)

The observation of these peaks in both regions indicates that the reaction is reversible, which is a desirable property in energy storage systems [38,39]. The intensities of these peaks are also influenced by ion adherence to the electrode surface and the scanning speed. Here, as the scanning speed increases, the intensities of these peaks are expected to increase in a linear direction as seen in previous studies [11,26]. This is confirmed to occur with a slope of 0.98942 in the anodic region and 0.99924 in the cathodic region in the Randel–Sevic plot of Figure 6c. On the other hand, the determination of the charging mechanism of such electrodes is an important issue, which can be clarified from the CV graph. For this, based on the $I_{pa}=a\times v^{b}$ formula, firstly the logarithm of the anodic peaks log (Ip) versus the scan rates log (V) is drawn, where a and b are variable constants and the b value is determined from the slope of this graph, as seen in Figure 6d [40]. Considering the slope of the linear line (b value) in this graph, the charging mechanism is primarily diffusion-controlled (or battery-type behavior) when it is close to 0.5, and capacitive-controlled when it is close to 1 [41]. Based on a b value of 0.75, the charging mechanism of the electrode in this case appears to be both capacitive and diffusion-controlled. A series of calculations were made according to Dunn’s method based on the formula *i(v) = k*_1_*v + k*_2_*V*^1/2^ [42], and the charging mechanism of this electrode was understood to be 34% capacitive and 66% diffusion controlled.

Another measurement needed to determine the performance of an electrode is galvanostatic charge–discharge (GCD). Chronoamperometry is used in this measurement to determine the charge and discharge times at various current densities. This makes it possible to calculate the specific capacity, energy and power density of the electrode at various current densities. The results of the GCD measurement performed for this purpose are shown in Figure 6b. According to these measurements, the discharge durations of the electrode at current densities of 2 mA, 3 mA, 4 mA, 5 mA, 6 mA, and 7 mA were determined to be 138 s, 84 s, 56 s, 40 s, 31 s and 25 s, respectively. Similarly, the specific capacity values at these currents were calculated as 525 mF cm^−2^, 480 mF cm^−2^, 426 mF cm^−2^, 380 mF cm^−2^, 354 mF cm^−2^ and 333 mF cm^−2^, as seen in Figure 6e. Additionally, the energy (E) densities of this electrode at the same currents were calculated as 8.9 mWh cm^−2^, 8.1 mWh cm^−2^, 7.25 mWh cm^−2^, 6.48 mWh cm^−2^, 6.02 mWh cm^−2^ and 5.67 mWh cm^−2^, while the power (P) densities were calculated as 233 mW cm^−2^, 350 mW cm^−2^, 466 mW cm^−2^, 583 mW cm^−2^ and 816 mW cm^−2^ as shown in Figure 6f. According to these results, as the current density increases, both specific capacity and energy density decrease. This situation is directly related to the electrode interaction of the ions [43]. While ions have the opportunity to enter the inner parts of the electrode at low currents, they quickly accumulate on the surface and form an ion layer at high currents and cannot reach the inner parts. This reduces the ion retention, i.e., energy storage, of the electrode.

**Figure 7:**
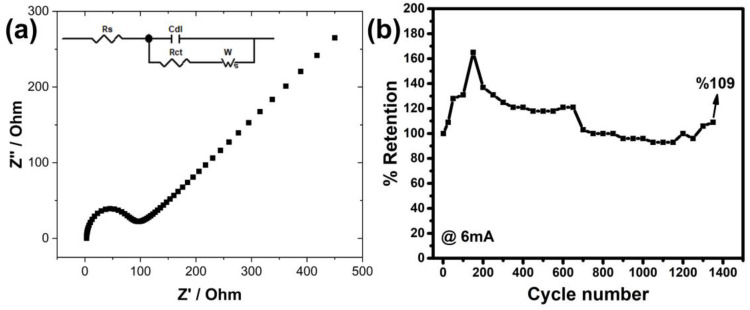
(**a**) EIS and (**b**) multiple charge–discharge (stability) measurements.

The electrochemical impedance (EIS) measurements given in Figure 7a were carried out to examine the interactions between the electrode and the electrolyte. As can be seen from the graph, there are two different regions from low high frequency to low frequency. The beginning of the semi-circle observed in the first of these expresses the resistance of the solution (Rs), while its diameter expresses the charge transfer resistance (Rct) between the electrode and electrolyte [44]. Here, it is understood that the resistance of the solution is about 2.9 ohm, and the charge transfer resistance is 52.4 ohm. These resistance values are lower compared to our previous pure CuFe_2_O_4_, charge transfer in particular [31]. The linear region of the graph is related to the diffusion resistance of the ions and is called the Warburg impedance (Zw) [45]. The Zw value is known according to the ${|Z}_{w}|$ = $\frac{\sqrt{2}\sigma }{\omega^{1/2}}$ formula [46], and the sigma value (σ) in the formula is related to the equation $Y_{0}=\frac{1}{\sqrt{2\sigma }}$ [47]. The Y_0_ value of the CuFe_2_O_4_ electrode produced in this study was measured as 92 Ω s^−0.5^, which means that the higher this value, the smaller the sigma, which means that the diffusion resistance is lower. This increases the capacity of the electrode, which was already a significant increase compared to our previous study.

Apart from these, the results of multiple charge–discharge measurements at a current of 6 mA, which were performed to test the stability of electrode, are shown as a % retention in the graph in Figure 7b. According to these results, the % retention values for the first 25, 50, 100 and 150 cycles reached 109, 128, 131 and 165%, respectively, with increasing discharge time. Then, the % retention values gradually decreased to 100% until the conversion amount reached 700. When the number of cycles continued up to 1350, the stability of the electrode was observed to be continued with ±10 increase/decrease. The stability value was seen to exceed 100% in both our earlier experiments and numerous other investigations that have been published. The reason for this is thought to be that the ions in the electrolyte reach more active cites of the electrode over time, but there is not enough time at the beginning of the cycle. This situation can actually be interpreted as an increase in the wetting of the electrode by the ions in the first cycles. Additionally, it is believed that the unstable electrode is similarly affected by variations in solution concentration in long-term measurements. This precarious condition was further made clearer by the graph, which shows the results of nearly every 25 cycles.

Table 2 summarizes the findings of the investigations that have been performed so far on CuFe_2_O_4_. According to these results, the effect of potential range, electrolyte type and other measurement parameters is evident. Even more important is the shape of the produced CuFe_2_O_4_. The capacity values of CuFe_2_O_4_ particles, which are spherical and have no other special shape, could reach almost 200 F g^−1^. Their stabilities also reached a maximum of 84%. However, the CuFe_2_O_4_ electrode developed by Bandgar et al. [48] in nanosheet form was the best ever, with 1501 F g^−1^ specific capacity and 98% stability after 10,000 cycles. From here, synthesis attempts in nanosheets or a similar geometry are likely to be successful. In our study, a cauliflower pattern similar to the CuFe_2_O_4_ sheet form was formed, which is the best performance after the study by Bandgar et al. with a specific capacity of 525 mF cm^−2^ and a stability of 109% after 1350 cycles. In fact, this value is approximately 250% better than CuFe_2_O_4_, which was synthesized in the form of a nano sponge in our previous study. This is probably because the structures with sheet geometry have a greater surface area and pore size. Another explanation is presumably the low wall thickness of these structures, because the thin ones have low interfacial resistance with the electrolyte, improving their ability to draw ions from the solution. On the other hand, the stability of an electrode after multiple cycles is related to the reversibility of the ions [49]. Depending on the geometry of the components that make up the electrode, the ion holding ability also changes [50]. Since 2D shaped components can hold ions in a larger area, the return of these ions to the solution is probably more seamless [51]. Therefore, although there are similar products in the studies in this table, the difference in their stability is probably related to the surface shape and surface area.

## 4. Conclusions

The production of electrodes for supercapacitor applications from chalcopyrite ore was successfully achieved via leaching and hydrothermal synthesis in the present study. Surface analyses revealed that a cauliflower-patterned CuFe_2_O_4_ structure with an average wall thickness of 23 nm was observable on the nickel foam surface. Electrochemical performances of CuFe_2_O_4_ doped Ni foam, which is intended to be used directly as an electrode, show that the highest specific capacity was measured as 525 mF cm^−2^ at 2 mA and energy (E) and power (P) densities were calculated as 8.9 mWh cm^−2^ and 233 mW cm^−2^, respectively. The charging mechanism of this electrode was also estimated to be controlled by capacity to a degree of 34% and diffusion to a degree of about 66%.

## Figures and Tables

**Table 1:** XRF results of the enriched chalcopyrite ore.

Sample	Compositions (wt%)								
	Cu	S	Fe	Zn	Si	Al	Co	Ca	As
Chalcopyrite	22.36	35.04	38.06	2.85	0.89	0.3	0.25	0.17	0.08

**Table 2:** Comparison of the performances of CuFe_2_O_4_ based electrodes in the literature.

Electrode Materials	Electrolyte	Potential (V)	Scan Rate/Current Density	Specific Capacity (Cs)	% Retention	Ref.
Pure CuFe_2_O_4_ nanosheets	6 M KOH	0 to 0.6	1 A g^−1^	1501 F g^−1^	~98% after 10,000 cycles at 10 A/g	[48]
Pure CuFe_2_O_4_ cauliflower	6 M KOH	0 to 0.35	2 mA	525 mF cm^−2^	~109% after 1350 cycles at 6 mA	This study
Pure CuFe_2_O_4_ nanoparticle	3 M KOH	0 to 0. 5	1 A g^−1^	~116 F g^−1^	~58% after 1000 cycles at 1 mA	[16]
Pure CuFe_2_O_4_	0.5 M H_2_SO_4_	0.15 to 0.75	0.004 V s^−1^	~73.6 F g^−1^	~52.1% after 2000 cycles at 1 V/s	[39]
Pure CuFe_2_O_4_ nanospherical	1 M KOH	0 to 0.5	0.5 A g^−1^	~189 F g^−1^	~84% after 1000 cycles at 3 A/g	[52]
Pure CuFe_2_O_4_ nanosponge	6 M KOH	0 to 0.45	2 mA	148 mF cm^−2^	-	[31]

## Data Availability

All the data is available in this manuscript.
